# Troubleshooting in Designing and Fabricating a Hollow Bulb Cast Partial Denture in a Partial Maxillectomy Patient: A Case Report

**DOI:** 10.7759/cureus.58220

**Published:** 2024-04-14

**Authors:** Madhavi Selukar, Surekha Godbole, Shubham U Tawade, Sharayu Nimonkar

**Affiliations:** 1 Prosthodontics, Sharad Pawar Dental College and Hospital, Datta Meghe Institute of Higher Education and Research, Wardha, IND

**Keywords:** mucormycosis, covid-19, components of cast partial denture, cast partial denture, hollow bulb obturator

## Abstract

The most common complication post COVID-19is mucormycosis. It is an opportunistic infection caused by the fungus mucormycetes which belongs to the order *Mucorales*. This infection can spread from the oral cavity to the nasal cavity and sometimes also involve the orbit. Surgical resection of the affected region followed by its prosthetic rehabilitation is one of the viable treatment options. In this case report, we will discuss the problems in designing and fabricating a hollow bulb obturator and its solutions.

## Introduction

Patients with maxillary defects with oronasal communication are vulnerable to develop fluid leakage into the nasal cavity, hypernasal speech, and impairment in mastication. In these cases, an obturator prosthesis can be planned to establish an oronasal seal [1]. The incidence and prevalence of mucormycosis have rapidly increased post COVID-19 pandemic. Mucormycosis also known as black fungus is an opportunistic fungal infection caused by the fungus mucormycetes. This infection spreads to the maxilla which is rich in vascularity. The spread of the fungal infection is rapid, leading to the partial or complete resection of the maxilla [2].

The obturator prosthesis extension is based on the configuration of the defect, mucosal lining of the tissue, functional requirements to establish stabilization, support, and retention of the prosthesis, defect size, number of remaining teeth, and bony structures remaining [3]. The patient's acceptance of the prosthesis is an influencing factor that is responsible for the fabrication of the prosthesis [4].

This case report addresses the problems associated with the "designing and fabrication of cast partial denture with a hollow bulb obturator prosthesis." The present case report also gives insight into solving the problems associated with the fabrication of the prosthesis.

## Case presentation

**Figure 1:**
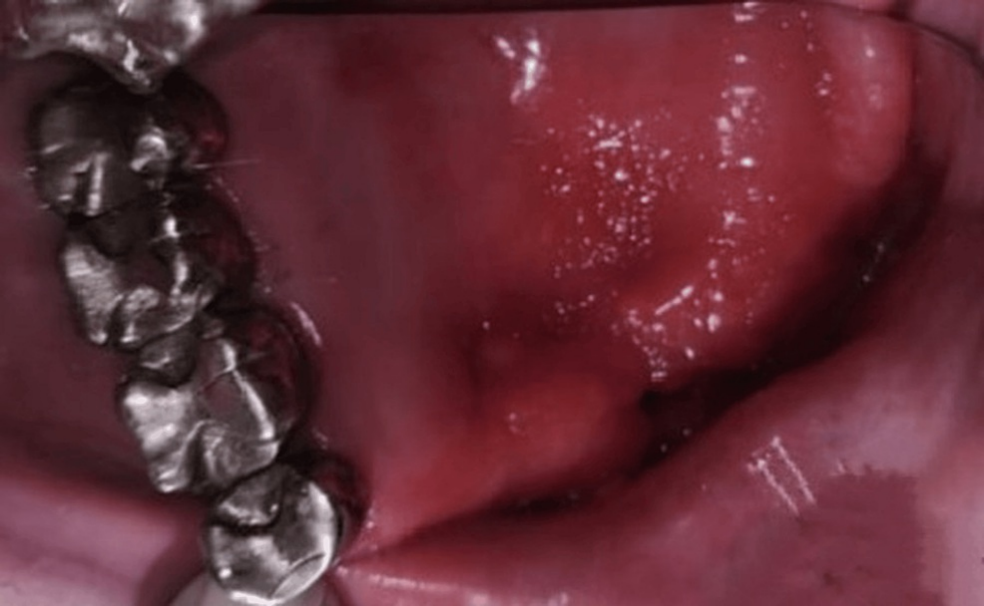
Intraoral photograph of the partial maxillectomy patient with Aramany's class IV defect.

A 48-year-old female patient reported to the Department of Prosthodontics and Crown and Bridge for the prosthetic rehabilitation of post-maxillectomy defect resulting from mucormycosis post COVID-19 infection. The patient was operated on for mucormycosis, five years back at a charitable trust hospital. The left quadrant of the maxilla was surgically resected. The defect was crossing the midline. On intraoral examination, the posterior stops that were present were 15, 16, 17, and 18. This defect can be classified as per Aramany's classification into class IV maxillary defect (Figure 1). 

**Figure 2:**
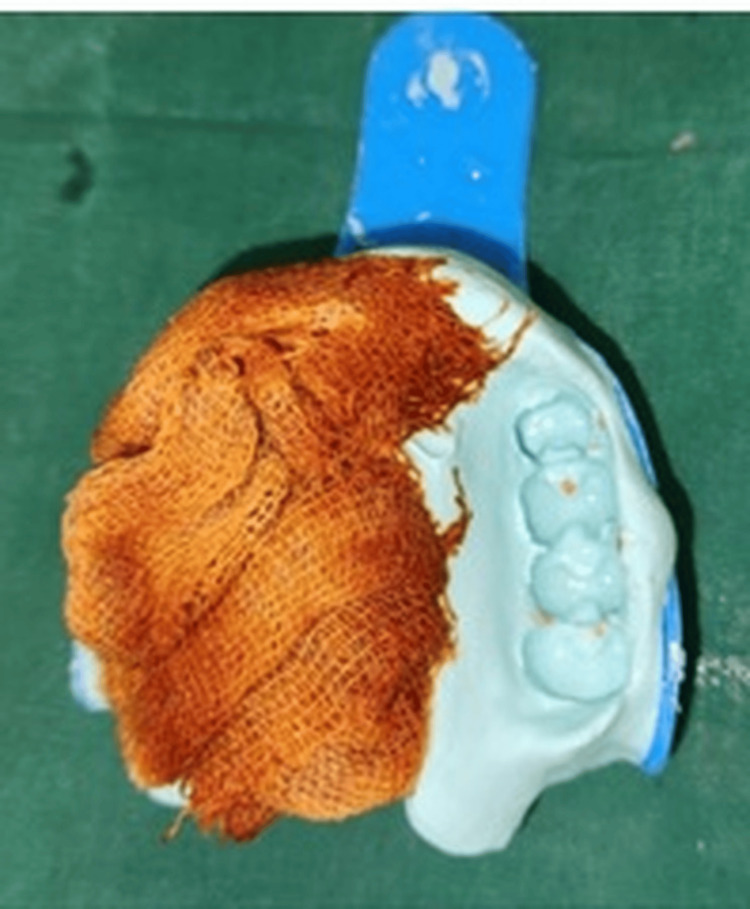
A preliminary impression made with irreversible hydrocolloid material (tropicagel).

The workflow for fabricating a cast partial denture with a hollow bulb obturator prosthesis was as follows. A primary impression was made with irreversible hydrocolloid material (Zhermack Tropicalgin Alginate) (Figure 2).

**Figure 3:**
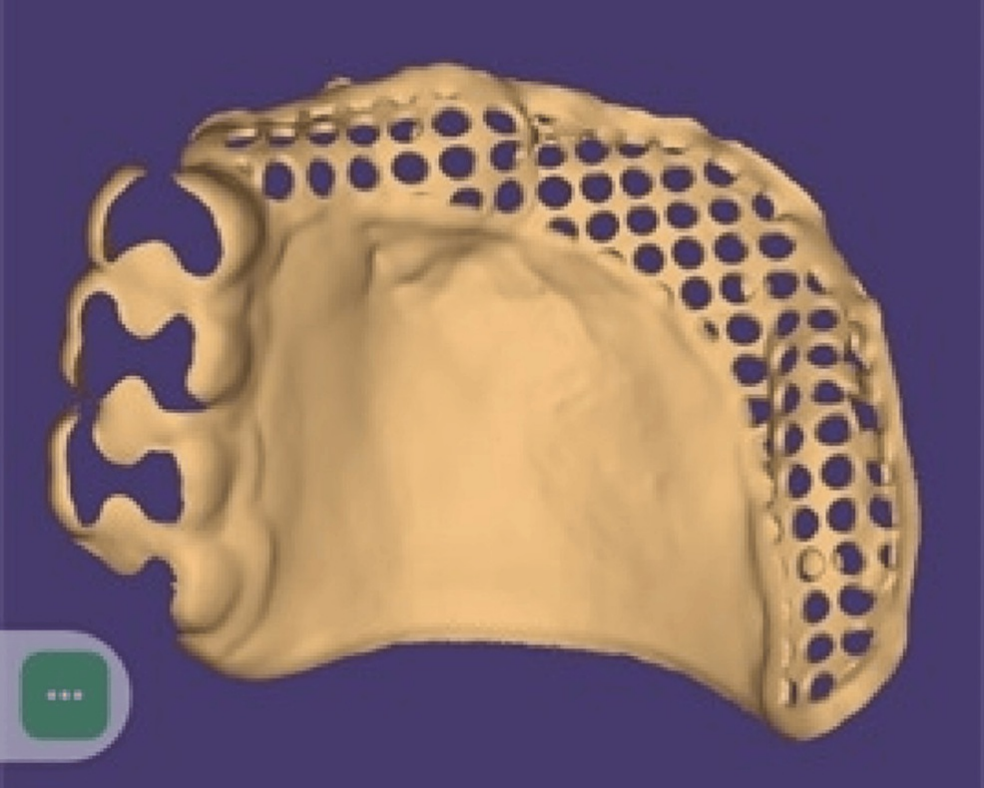
Designing of the metal framework. Designing of metal framework done through the exocad software.

A type 2 gypsum which is a dental plaster was used to pour the diagnostic impression and to obtain a diagnostic cast. The said diagnostic cast was surveyed. A tentative jaw relation record was made. Case planning mouth preparation was done. Teeth 15, 16, 17, and 18 were prepared for the rest, rest seats were incorporated in the prepared tooth, and an elastomeric impression material (Zhermack Zetaplus) was used to make the impression. The fabricated crowns with internal rest were cemented. Spacer wax was adapted, and a special tray was fabricated with a tray material that is 2 mm short from the sulcus depth. The special tray was checked intraorally for extension, border moulding was done with a green stick compound, and a wash impression was made with a light body impression material. A functional impression was taken with irreversible hydrocolloid impression material (tropicagel). The technique for recording functional impressions was Hindle's modification of McLean's technique. A finished polished record base was made, and occlusal rims were adapted. A jaw relation record was made. Designing of the metal framework was done through the exocad software, and the metal framework was milled using computer-aided designing/computer-aided manufacturing (CAD/CAM) as shown in Figure 3. 

A jaw relation record was made, the teeth were arranged, and the try-in was done. The impression was sent to a laboratory for the fabrication of a hollow bulb obturator. A preformed ice block was used to make the hollow bulb obturator. The flasking procedure was carried out, and the finishing and polishing of the denture were done followed by the insertion of the prosthesis.

**Figure 4:**
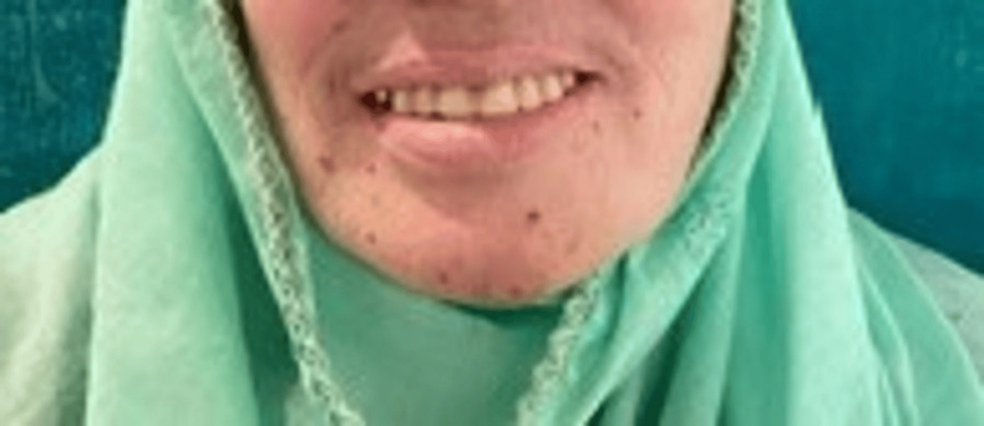
Post-insertion photograph of the patient.

The patient was happy and satisfied with the results. A post-insertion photograph of the patient during the three-month follow-up was taken (Figure 4). 

## Discussion

Singh et al. commented that the maxillectomy defects resulted in oroantral communication. This communication caused discomfort to the patient during chewing and swallowing, impaired speech, and facial disfigurement. They described a method of fabricating a definitive obturator with a cast metal framework using a single flask and a one-time processing method for an acquired maxillary defect. Following the designing principles, a tripodal design was selected by the authors. This ensured a maximum distribution of the functional load to the tissue. The right first premolar was used for indirect retention. Direct retention was given by the "I-bar clasp" and "circumferential clasp" on the left and right first premolars, respectively, and the "embrasure clasp" between the right first and second molars [1].

Nimonkar et al. have reviewed different techniques to fabricate a hollow bulb obturator, the advantages and disadvantages of every technique, and their methods of fabrication. Fifteen methods were reviewed in this article for the fabrication of a hollow bulb obturator. The most user-friendly method is the fabrication of a hollow bulb obturator using an ice cube [2].

Singh et al. stated that the obturator prosthesis is most common for the effective rehabilitation of oronasal defects. In cases of large maxillary defects, rotation of the prosthesis takes place to counteract this defect, and an indirect retainer is used. Surgical intervention is needed to treat malignancy; this will create a connection between the oral cavity, nasal cavity, and maxillary sinus. In these cases, the patient faces difficulty in performing normal functions. An obturator prosthesis restores the missing structures and establishes communication among the various cavities [3].

Problems associated with the fabrication of the prosthesis

When designing a hollow bulb obturator, some potential problems to consider include ensuring proper fit and comfort for the patient, preventing leakage of fluids or air, maintaining the durability and longevity of the device, and addressing any potential issues with cleaning and maintenance. It's important to carefully consider these factors to create an effective and reliable product for the intended use [5-7].

Table 1 depicts the troubleshooting in designing and fabricating a hollow bulb cast partial denture and their associated solutions (Table 1) [8-10].

**Table 1:** Troubleshooting in designing and fabricating a hollow bulb cast partial denture and their associated solutions.

Sr. no.	Clinical/laboratory step	Problem associated	Solution
1	Making of preliminary and functional impression	In conditions when the patient is apprehensive. When the mucosa is sensitive, there are no bony structures present. Inelasticity of surrounding musculature. Patient with dry mouth. In conditions where the patient aspirates the material.	Patient education and treatment planning are the most important factors to be considered. A clinician should try to make the patient relaxed. The patient should be explained about the treatment procedure. Proper selection of a tray should be done before making an impression. An overextended tray may cause trauma to the mucosa. An underextended tray will not record the depth of the tissue surface. The condition of dry mouth is addressed by sialogogue. Proper chair position can solve problems like aspiration of material while recording the impression.
2	Metal framework try-in	Rest seats are in supra-occlusion. The metal framework is overextended and heavy.	Trimming and adjustment of the metal framework should be done.
3	Jaw relation	Can't in the maxillary occlusal plane.	Adjustment of the maxillary occlusal plane.
4	Try-in	Increased lip fullness and proclined anterior teeth.	Decrease the lip fullness by reducing the wax rim and adjust the anterior teeth by slightly making it retroclined.
5	Flasking and dewaxing procedures	Residual wax remaining after dewaxing.	During the flasking procedure after base flasking, the procedure called coring is done to prevent the breaking of teeth. Place the flask in running hot tap water to remove residual wax.
6	Packing of hollow bulb obturator	Porosity.	In the fabrication of a hollow bulb obturator, an ice cube is used to make it hollow. The incorporation of an ice cube may create porosity in the denture. To overcome this, paint a mixture of clear acrylic and cold cure on the buccal side and ask the patient to make whistling, smiling, and speaking movements so that the modiolus area will be recorded. This will aid in increased retention.
7	Denture insertion	Overextended posterior and buccal borders.	Mark the overextended borders and trim them.

## Conclusions

The fabrication of a cast partial denture with a hollow bulb obturator is the most convenient treatment option for the patient post mucormycosis for partial maxillectomy patients. This treatment option is economical and less time-consuming; also, there is no need to undergo any further surgical procedure. 
